# Human Papillomavirus Vaccination by Birth Fiscal Year in Japan

**DOI:** 10.1001/jamanetworkopen.2024.22513

**Published:** 2024-07-16

**Authors:** Asami Yagi, Yutaka Ueda, Emiko Oka, Satoshi Nakagawa, Tadashi Kimura

**Affiliations:** 1 Department of Obstetrics and Gynecology, Osaka University Graduate School of Medicine, Osaka, Japan

## Abstract

**Importance:**

The Ministry of Health, Labour, and Welfare (MHLW) of Japan aggregates human papillomavirus (HPV) vaccination data across Japan for each fiscal year (FY) by age at vaccination. Birth FY (BFY)–specific vaccination coverage remains unknown.

**Objective:**

To calculate the BFY-specific vaccination coverage for each FY and the cumulative first-dose coverage for each BFY in Japan, to understand the generation-specific vaccination coverage, and to estimate the cumulative first-dose coverage of each BFY that would be achieved by FY 2028 vs World Health Organization (WHO) targets.

**Design, Setting, and Participants:**

In this cross-sectional study, MHLW-published national age-specific HPV vaccination numbers and demographic data for female individuals were used to calculate the BFY-specific first-dose coverage for each FY and the BFY-specific cumulative first-dose coverage. It was assumed that the BFYs 2007 to 2012 vaccination coverage in FY 2023 to 2028 would remain the same as the vaccination coverage of the same grade in FY 2022 to estimate the cumulative first-dose coverage that would be achieved by FY 2028. Data analysis was performed from December 2023 to January 2024.

**Exposure:**

Two MHLW policy changes were the government’s suspension of proactive recommendation for HPV vaccination in June 2013 and the government’s resumption of proactive recommendation for HPV vaccination in April 2022.

**Main Outcomes and Measures:**

The primary outcome was generation-specific vaccination coverage among female individuals born in BFYs 1994 to 2010 in FYs 2010 to 2022, calculated using reconfigured published data.

**Results:**

In this study of vaccination data for 9 414 620 female individuals, the generation-specific vaccination coverage was 71.96% for the vaccination generation (BFYs 1994-1999), 4.62% for the vaccine-suspension generation (BFYs 2000-2003), 16.16% for the generation that received information individually (BFYs 2004-2009), and 2.83% for the vaccine-resumed generation (BFY 2010). HPV routine vaccination coverage was extremely low in BFYs 2000 to 2010 (0.84%-25.21%) vs BFYs 1994 to 1999 (53.31%-79.47%). The cumulative first-dose coverage that was estimated to be achieved in the vaccine-resumed generation by FY 2028 plateaued at 43.16%.

**Conclusions and Relevance:**

Even after the resumption of MHLW’s proactive recommendations, HPV vaccination coverage has only minimally recovered in Japan. The cumulative first-dose coverage that was estimated to be achieved in the vaccine-resumed generation by FY 2028 is below the WHO target. These findings reveal that stronger cervical cancer control measures are required, particularly for the vaccine-resumed generation, which will plateau at approximately one-half the WHO target values.

## Key Points

**Question:**

How has Japan’s human papillomavirus (HPV) vaccination coverage recovered after resumption of the government’s proactive recommendation?

**Findings:**

This cross-sectional study using reconfigured published data for 9 414 620 female individuals found that HPV routine vaccination coverage was extremely low in birth fiscal years (BFYs) 2000 to 2010 (0.84%-25.21%) vs BFYs 1994 to 1999 (53.31%-79.47%). Assuming that the vaccination coverage in FYs 2023 to 2028 would remain the same as FY 2022, the cumulative coverage would plateau at 43.16%.

**Meaning:**

These findings suggest that HPV vaccination coverage in Japan is extremely low and is estimated to fall short of the World Health Organization target in FY 2028.

## Teaser

This cross-sectional study evaluates human papillomavirus vaccination coverage in Japan from 1994 to 2010 to estimate the cumulative first-dose coverage that would be achieved by 2028 vs World Health Organization targets.

## Introduction

In 2020, the World Health Organization (WHO) launched its strategy for cervical cancer elimination, with a goal for all countries to achieve human papillomavirus (HPV) vaccination coverage of 90% by 2030.^1^ In 2022, WHO released its estimate of first-dose vaccination coverage among female individuals by the age of 15 years in 91 countries, with 15 countries, including Uganda and Rwanda achieving vaccination coverage of 90% or higher.^2^

In the context of HPV vaccination among the world’s high-income nations, Japan is in a critical situation. In Japan, a public subsidy program for HPV vaccine for female individuals in grades 7 to 11 began in fiscal year (FY) 2010,^3^ and HPV vaccination is not yet a national routine vaccination, as defined by Japan’s Immunization Act. However, almost-free HPV vaccinations are available using public subsidies from local governments. The initial target population was female individuals born in FY 1994 to 1997. In April of FY 2013, the Immunization Act adopted HPV vaccination for female individuals in grades 6 to 10 (birth FYs [BFYs] 1997-2001) as part of its national routine vaccination program.^4^ However, within months, media reports of potentially serious, diverse symptoms after vaccination surfaced. In June of FY 2013, Japan’s Ministry of Health, Labour, and Welfare (MHLW) announced a temporary suspension of its proactive recommendation for the HPV vaccine,^5,6^ leading to an immediate and dramatic decline in the public’s trust in and uptake of the HPV vaccine.^7,8,9,10,11^ Larson et al^11^ reported that the longer Japan’s recommendation suspension continued, the more the public’s concern grew. The WHO Global Advisory Committee on Vaccine Safety commented on the situation in Japan in their 2015 Statement on the Safety of HPV vaccines, which reported that Japan was in a situation where, as a result of government choices, “young women are being left vulnerable to HPV-related cancers that otherwise could be prevented” and “policy decisions based on weak evidence, leading to lack of use of safe and effective vaccines, can result in real harm.”^12^

The vaccine was highly effective in preventing HPV-caused precancerous cervical lesions^13,14,15,16,17,18^ and invasive cervical cancers.^19,20^ Two epidemiological studies^21,22^ of HPV vaccine safety conducted in Japan demonstrated that the HPV vaccine was consistently safe. Many reports^23,24,25,26,27^ from overseas have also supported the safety of the HPV vaccine. Therefore, considering such data, MHLW recommended that local governments provide HPV vaccine information by mail individually to people eligible for regular vaccinations from FY 2020.^28,29^ In November of FY 2021, the MHLW finally decided to restart its proactive recommendation for the HPV vaccine, which was actually resumed in FY 2022.^30^ In April FY 2022, Japan also started a catch-up vaccination program targeting BFYs 1997 to 2007.^31^ The program was time-limited to 3 years, from FY 2022 to FY 2024.^32^ However, the real harm indicated by the WHO was confirmed by increased HPV 16/18 infection rates and abnormal cervical cytology rates due to reduced vaccination in BFY 2000.^33,34^

This study primarily aimed to calculate the BFY-specific vaccination coverage and cumulative vaccination coverage for each BFY in Japan, where HPV vaccine policies have changed greatly. Furthermore, we determined the generation-specific vaccination coverage resulting from differences in the vaccination environment (vaccination generation, BFYs 1994-1999; the vaccine-suspension generation, BFYs 2000-2003; the generation that received information individually, BFYs 2004-2009; and the vaccine-resumed generation, BFY 2010). The secondary objective of this study is to estimate the cumulative vaccination coverage of each BFY to be achieved by FY 2028 and to compare this value with the WHO target value.

## Methods

### Study Design and Setting

This cross-sectional study was approved by the Ethics Committee of the Osaka University Hospital. Informed consent was not needed because the data were publicly available, in accordance with 45 CFR §46. This study followed the Strengthening the Reporting of Observational Studies in Epidemiology (STROBE) reporting guideline. This study used national HPV vaccination numbers by age at vaccination and demographic data.^35,36,37,38,39,40^

### Outcomes

This descriptive epidemiological study analyzed BFY-specific cumulative first-dose coverage and generation-specific vaccination coverage resulting from differences in vaccination environment among female individuals from BFYs 1994 to 2010 in FY 2010 to 2022. Furthermore, we estimated the cumulative first-dose coverage for BFYs 2007 to 2012 to be achieved by FY 2028. In Japan, the Order for Enforcement of the Immunization Act sets vaccination targets for each BFY. However, the MHLW only publishes the national figure for the number of people vaccinated by age at vaccination, and the BFY-specific vaccination coverage for each FY and cumulative vaccination coverage for each BFY were unclear. Understanding these values was necessary for evaluating the impacts of the 2 MHLW policy changes on the HPV vaccination environment for Japanese women (the government’s withholding of proactive recommendation for HPV vaccination in June 2013 and the government’s resumption of proactive recommendation for HPV vaccination in April 2022). In Japan, the fiscal year starts in April and ends in March of the following year (eFigure 1 in Supplement 1).

### Data Sources

We obtained vaccination data released by the MHLW regarding routine HPV vaccinations with the first dose by age at vaccination (not by BFY) from FY 2010 to FY 2022 (eTable 1 in Supplement 1). These reports included the implementation report of the Emergency Promotion Project of HPV vaccination from FY 2010 to FY 2012,^35^ regional health promotion program data from FY 2013 to FY 2021,^36,37^ and the preliminary FY 2022 monthly vaccination counts released by the MHLW.^38^ The Emergency Promotion Project of HPV Vaccination was a local government–led program that was conducted using funds from this project.^39^ The number of female individuals targeted for the HPV vaccine was based on population data from the national population census.^40^ In this study, HPV vaccination recipients were defined as female individuals who received the HPV vaccine with a public subsidy or as a routine vaccination, regardless of the type of vaccine.

### Statistical Analysis

#### Cumulative Routine HPV Vaccination With First-Dose Coverage Through FY 2022 by BFY

Data analysis was performed from December 2023 to January 2024. We reconfigured the number of HPV vaccinations, which was initially reported as first dose by age into the first dose by BFY. There is no one-to-one correspondence between age at vaccination and BFY. One age at vaccination may have 2 BFY values (eTable 2 in Supplement 1). For example, a 13-year-old at the time of vaccination in FY 2022 may be in either 7th or 8th grade (ie, BFY 2009 or BFY 2008). The methods used for these calculations were the same as those used previously.^41,42^ However, we reconfigured the number of HPV first-dose vaccinations for BFY in FY 2022 using a new method for greater accuracy, as shown in Table 1. The number of vaccinations for each month was recalculated from age-specific values to BFY-specific values. For example, the vaccination ages were 11 and 12 years for BFY 2010 (6th grade) and 12 and 13 years for BFY 2009 (7th grade). Therefore, the number of vaccinations at the age of 12 years for each month was divided by 12 and distributed to BFY 2009 and BFY 2010, respectively, from April, the month in which the FY began, to March, the month in which the FY ended. However, the number of monthly vaccinations for 11-year-olds was assumed to be BFY 2010 (6th grade), and the number of monthly vaccinations for 16-year-olds was assumed to be BFY 2006 (10th grade). We combined these coverages to reveal the cumulative routine vaccination coverage with a first dose for each BFY from 1994 to 2010 for the period FY 2010 to FY 2022.

**Table 1.** Method for Reconfiguring the Number of Routine Human Papillomavirus Vaccinations With a First Dose From Month in Fiscal Year 2022 to Birth Fiscal Year^a^

Birth fiscal year (grade)	April	May	June	July	August	September	October	November	December	January	February	March
2010 (6th grade)	11 y + 12 y × 0/12	11 y + 12 y × 1/12	11 y + 12 y × 2/12	11 y + 12 y × 3/12	11 y + 12 y × 4/12	11 y + 12 y × 5/12	11 y + 12 y × 6/12	11 y + 12 y × 7/12	11 y + 12 y × 8/12	11 y + 12 y × 9/12	11 y + 12 y × 10/12	11 y + 12 y × 11/12
2009 (7th grade)	12 y × 12/12 + 13 y × 0/12	12 y × 11/12 + 13 y × 1/12	12 y × 10/12 + 13 y × 2/12	12 y × 9/12 + 13 y × 3/12	12 y × 8/12 + 13 y × 4/12	12 y × 7/12 + 13 y × 5/12	12 y × 6/12 + 13 y × 6/12	12 y × 5/12 + 13 y × 7/12	12 y × 4/12 + 13 y × 8/12	12 y × 3/12 + 13 y × 9/12	12 y × 2/12 + 13 y × 10/12	12 y × 1/12 + 13 y × 11/12
2008 (8th grade)	13 y × 12/12 + 14 y × 0/12	13 y × 11/12 + 14 y × 1/12	13 y × 10/12 + 14 y × 2/12	13 y × 9/12 + 14 y × 3/12	13 y × 8/12 + 14 y × 4/12	13 y × 7/12 + 14 y × 5/12	13 y × 6/12 + 14 y × 6/12	13 y × 5/12 + 14 y × 7/12	13 y × 4/12 + 14 y × 8/12	13 y × 3/12 + 14 y × 9/12	13 y × 2/12 + 14 y × 10/12	13 y × 1/12 + 14 y × 11/12
2007 (9th grade)	14 y × 12/12 + 15 y × 0/12	14 y × 11/12 + 15 y × 1/12	14 y × 10/12 + 15 y × 2/12	14 y × 9/12 + 15 y × 3/12	14 y × 8/12 + 15 y × 4/12	14 y × 7/12 + 15 y × 5/12	14 y × 6/12 + 15 y × 6/12	14 y × 5/12 + 15 y × 7/12	14 y × 4/12 + 15 y × 8/12	14 y × 3/12 +15 y × 9/12	14 y × 2/12 + 15 y × 10/12	14 y × 1/12 + 15 y × 11/12
2006 (10th grade)	15 y × 12/12 + 16 y	15 y × 11/12 + 16 y	15 y × 10/12 + 16 y	15 y × 9/12 + 16 y	15 y × 8/12 + 16 y	15 y × 7/12 + 16 y	15 y × 6/12 + 16 y	15 y × 5/12 + 16 y	15 y × 4/12 + 16 y	15 y × 3/12 + 16 y	15 y × 2/12 + 16 y	15 y × 1/12 + 16 y

^a^ Years refer to ages at vaccination.

The 95% CIs were obtained using the 2-sided Agresti-Coull method for determining confidence limits for a binomial proportion.^43^ STATA MP statistical software version 18 (StataCorp) was used for statistical analyses. When no overlap in the 95% CIs was observed, the difference was regarded to be statistically significant.

#### Estimated Cumulative Routine HPV Vaccination With First-Dose Coverage Through FY 2028

We clarified the extent of HPV vaccination coverage if the vaccination dissemination status in FY 2022 continued in FY 2023 to 2028 by assuming that the vaccination coverage in FY 2023 to 2028 for those born in BFYs 2007 to 2012 will remain the same as the vaccination coverage for the same grade in FY 2022. We estimated the cumulative first-dose coverage to be achieved by FY 2028 for people born in BFYs 2007 to 2012. In addition, under the same assumption, we calculated the cumulative vaccination coverage until FY 2028 using increments of 1.2, 1.1, 0.9, and 0.8 multiples of the current vaccination coverage for each BFY.

## Results

### Cumulative Routine HPV Vaccination With First-Dose Coverage Through FY 2022 by BFY

**Figure 1.**
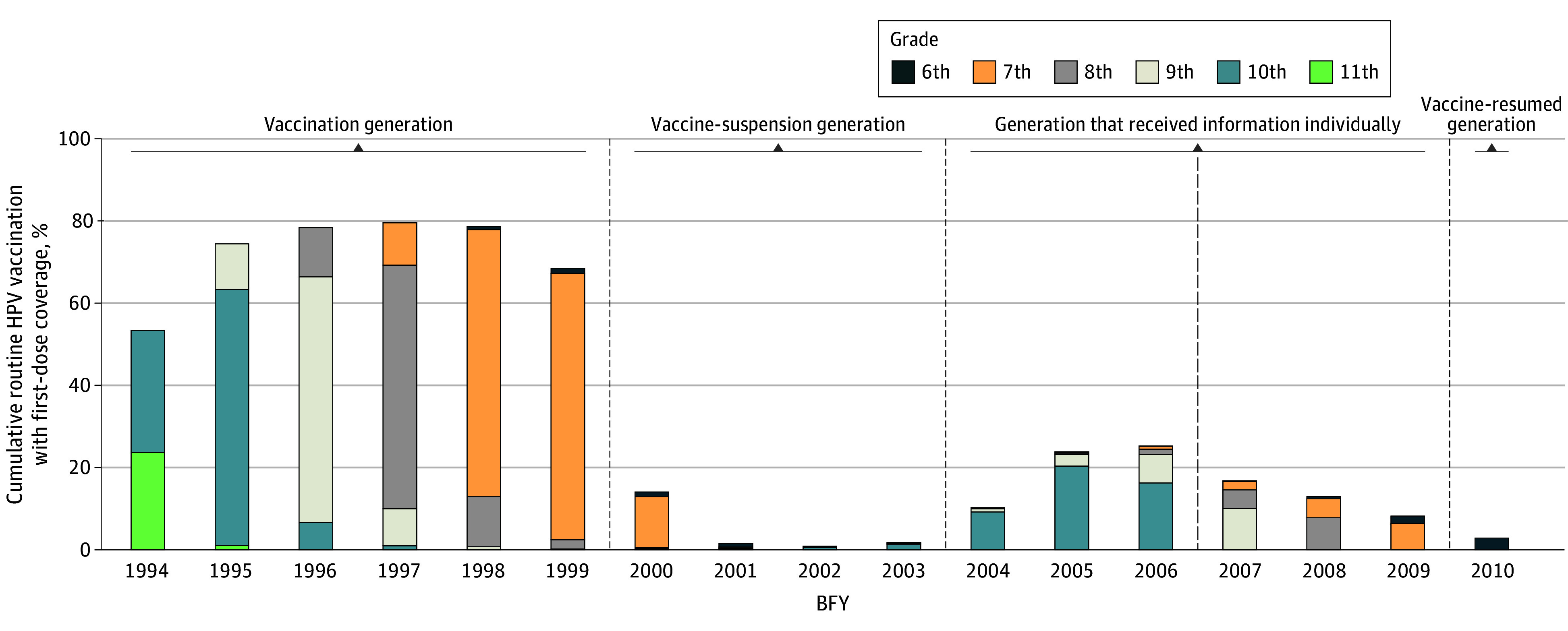
Cumulative Routine Human Papillomavirus (HPV) Vaccination With First-Dose Coverage Through Fiscal Year (FY) 2022 by Birth Fiscal Year (BFY) The vaccination generation is BFYs 1994 to 1999 (cumulative vaccination rate, 71.96%), the vaccine-suspension generation is BFYs 2000 to 2003 (cumulative vaccination rate, 4.62%), the generation that received information individually is BFYs 2004 to 2009 (cumulative vaccination rate, 16.16%), and the vaccine-resumed generation is BFY 2010 (cumulative vaccination rate, 2.83%). BFYs 1994 to 2006 represent the targeted range for routine vaccination that ended in and before FY 2022. BFYs 2007 to 2010 represent targets for routine vaccination after FY 2022. Age ranges for grades are as follows: 11th grade, 16 to 17 years; 10th grade, 15 to 16 years; 9th grade, 14 to 15 years old; 8th grade, 13 to 14; 7th grade, 12 to 13 years; and 6th grade, 11 to 12 years old.

Vaccination data were available for a total of 9 414 620 female individuals. The cumulative routine HPV vaccination coverage with the first dose from FY 2010 to FY 2022 by BFY is shown in Table 2 and Figure 1. The vaccination coverage for the vaccination generation (BFYs 1994-1999) was 53.31% (95% CI, 53.18%-53.43%) to 79.47% (95% CI, 79.36%-79.57%).

**Table 2.** Reconfiguring Routine HPV Vaccination Coverage From the Age at Vaccination to Birth Fiscal Year

Grade^a^	Vaccination coverage, routine HPV vaccinations with a first dose, No. (%)																
	Vaccination generation^b^						Vaccine-suspension generation^c^				Generation that received information individually^d^						Vaccine-resumed generation^e^
	1994	1995	1996	1997	1998	1999	2000	2001	2002	2003	2004	2005	2006	2007	2008	2009	2010
11th	142 613 (23.65)	5855 (1.01)	NA	NA	NA	NA	NA	NA	NA	NA	NA	NA	NA	NA	NA	NA	NA
10th	178 852 (29.66)	362 112 (62.27)	39 224 (6.66)	5722 (0.98)	272 (0.05)	256 (0.04)	560 (0.10)	1366 (0.24)	2614 (0.47)	6774 (1.24)	49 015 (9.18)	104 516 (20.31)	85 413 (16.21)	NA	NA	NA	NA
9th	NA	64 387 (11.07)	351 268 (59.65)	52 608 (9.00)	3838 (0.66)	614 (0.11)	1112 (0.19)	534 (0.09)	800 (0.14)	1668 (0.31)	3944 (0.74)	14 364 (2.79)	36 634 (6.95)	52 802 (10.03)	NA	NA	NA
8th	NA	NA	70 362 (11.95)	345 946 (59.19)	71 102 (12.14)	13 198 (2.28)	1344 (0.23)	294 (0.05)	154 (0.03)	384 (0.07)	792 (0.15)	1838 (0.36)	6514 (1.24)	23 804 (4.52)	40 613 (7.74)	NA	NA
7th	NA	NA	NA	60 149 (10.29)	380 434 (64.95)	374 526 (64.74)	70 888 (12.34)	1413 (0.25)	857 (0.15)	490 (0.09)	663 (0.12)	1334 (0.26)	3833 (0.73)	10 644 (2.02)	24 082 (4.59)	32 710 (6.35)	NA
6th	NA	NA	NA	NA	4364 (0.75)	6836 (1.18)	6746 (1.17)	5010 (0.89)	236 (0.04)	178 (0.03)	96 (0.02)	134 (0.03)	402 (0.08)	908 (0.17)	3198 (0.61)	9438 (1.83)	14 494 (2.83)
Total	321 465 (53.31)	432 354 (74.36)	460 854 (78.25)	464 425 (79.47)	460 010 (78.53)	395 430 (68.36)	80 650 (14.04)	8617 (1.53)	4661 (0.84)	9494 (1.74)	54 510 (10.20)	122 186 (23.74)	132 796 (25.21)	88 158 (16.75)	67 893 (12.94)	42 148 (8.18)	14 494 (2.83)

Abbreviations: HPV, human papillomavirus; NA, not applicable.

^a^ Age ranges for grades are as follows: 11th grade, 16 to 17 years; 10th grade, 15 to 16 years; 9th grade, 14 to 15 years old; 8th grade, 13 to 14; 7th grade, 12 to 13 years; and 6th grade, 11 to 12 years old.

^b^ Birth fiscal yearly totals are as follows: 1994, 602 982 participants; 1995, 581 474 participants; 1996, 588 922 participants; 1997, 584 438 participants; 1998, 585 744 participants; and 1999, 578 486 participants. The total vaccination rate for this generation is 2 534 538 of 3 522 046 participants (71.96%; 95% CI, 71.92%-72.01%).

^c^ Birth fiscal yearly totals are as follows: 2000, 574 526 participants; 2001, 563 968 participants; 2002, 554 096 participants; 2003, 544 934 participants. The total vaccination rate for this generation is 103 422 of 2 237 524 participants (4.62%; 95% CI, 4.59%-4.65%).

^d^ Birth fiscal yearly totals are as follows: 2004, 534 211 participants; 2005, 514 615 participants; 2006, 526 834 participants; 2007, 526 445 participants; 2008, 524 881 participants; and 2009, 515 115 participants. The total vaccination rate for this generation is 507 691 of 3 142 101 participants (16.16%; 95% CI, 16.12%-16.20%).

^e^ Birth fiscal yearly total is 512 949 for 2010. The total vaccination rate for this generation is 14 494 of 512 949 participants (2.83%; 95% CI, 2.78%-2.87%).

The vaccination coverage for BFYs 2000 to 2010 was 0.84% (95% CI, 0.82%-0.87%) to 25.21% (95% CI, 25.10%-25.32%). The vaccine-suspension generation included the female individuals with BFYs 2000 to 2003. Notably, the HPV vaccination coverages in the vaccine-suspension generation were very low: 14.04% (95% CI, 13.95%-14.13%) for BFY 2000, 1.53% (95% CI, 1.50%-1.56%) for BFY 2001, 0.84% (95% CI, 0.82%-0.87%) for BFY 2002, and 1.74% (95% CI, 1.71%-1.78%) for BFY 2003.

The generation with BFYs 2004 to 2009 received information individually starting in FY 2020. Notably, vaccination coverage remained low compared with the vaccination generation. Coverage was 10.20% (95% CI, 10.12%-10.29%) for BFY 2004, 23.74% (95% CI, 23.63%-23.86%) for BFY 2005, and 25.21% (95% CI, 25.10%-25.32%) for BFY 2006, and it reached the end of their routine vaccination target age by FY 2022. The cumulative vaccination coverage for BFYs 2007 to 2009 did not reach 20% by the end of our study period, but they were still eligible for vaccination in FY 2023 and thereafter beyond.

Female individuals with BFY 2010 became age-eligible for routine vaccinations in FY 2022 when the government resumed its HPV vaccination recommendation (Figure 1). The first-year coverage was 2.83% (95% CI, 2.78%-2.87%), which was higher than that of the 12-year-olds of the vaccine-suspension generation and the generation that received information individually.

Compared with the WHO goal (for all countries to achieve an HPV vaccination coverage of 90%), the mean cumulative immunization coverage of the vaccination generation was 71.96% (95% CI, 71.92%-72.01%), which was the closest to the WHO goal of any group we studied. By FY 2022, the end point of this study, the vaccination coverage of the vaccine-suspension generation was 4.62% (95% CI, 4.59%-4.65%), that of the generation that received information individually was 16.16% (95% CI, 16.12%-16.20%), and that of the vaccine-resumed generation was 2.83% (95% CI, 2.78%-2.87%). The vaccination coverage of the vaccine-suspension generation (4.62%; 95% CI, 4.59%-4.65%) was significantly lower than that of the vaccination generation (71.96%; 95% CI, 71.92%-72.01%). In addition, the vaccination coverage of the generation that received information individually was significantly higher than that of the vaccine-suspension generation but was significantly lower than that of the vaccination generation. The vaccination coverage of the vaccine-resumed generation was significantly lower than that of the other 3 generations, but this generation still has a routine vaccination target period.

### Estimated Cumulative Routine HPV Vaccination With First-Dose Coverage Through FY 2028

**Figure 2.**
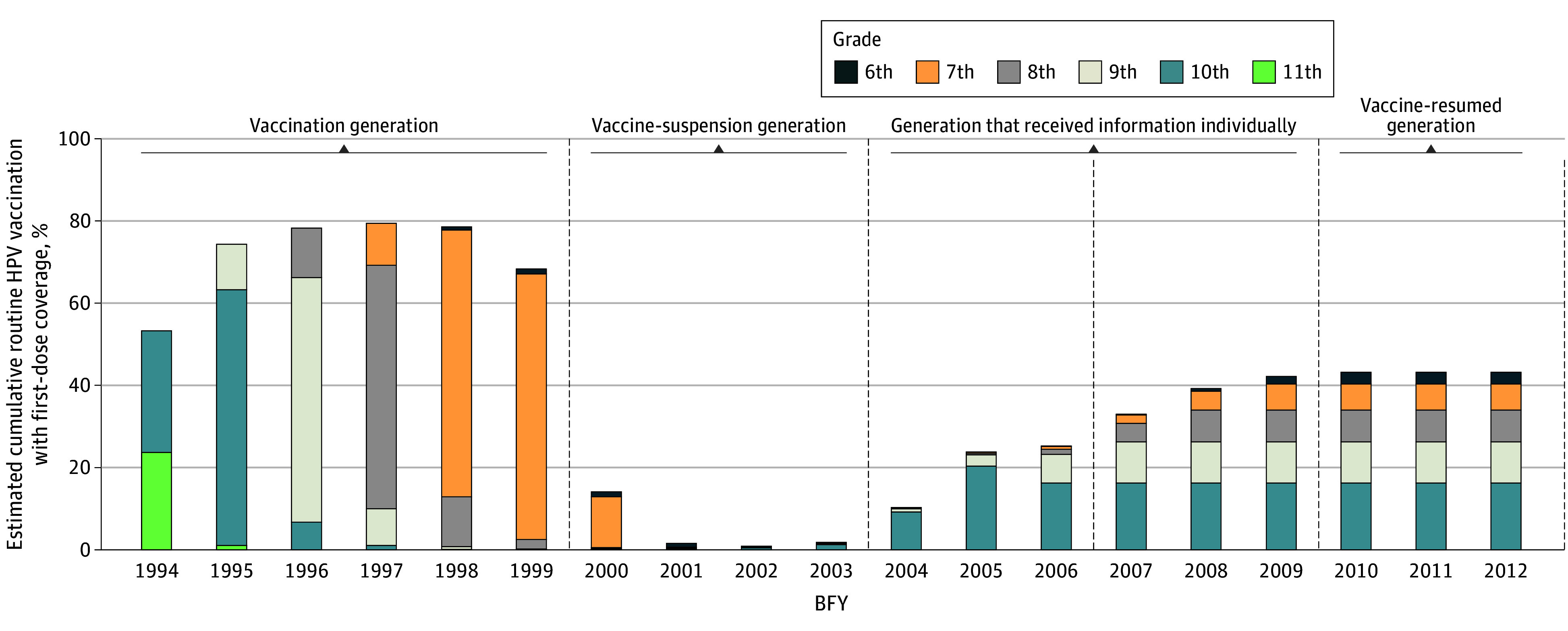
Estimated Cumulative Routine Human Papillomavirus (HPV) Vaccination With First-Dose Coverage Through Fiscal Year (FY) 2028 The vaccination generation is birth FYs (BFYs) 1994 to 1999 (cumulative vaccination rate, 71.96%), the vaccine-suspension generation is BFYs 2000 to 2003 (cumulative vaccination rate, 4.62%), the generation that received information individually is BFYs 2004 to 2009 (cumulative vaccination rate, 28.83%), and the vaccine-resumed generation is BFYs 2010 to 2012 (cumulative vaccination rate, 43.16%). BFYs 1994 to 2006 represent the targeted range for routine vaccination that ended in and before FY 2022. BFYs 2007 to 2012 represent targets for routine vaccination after FY 2022, which will end in and before FY 2028. Age ranges for grades are as follows: 11th grade, 16 to 17 years; 10th grade, 15 to 16 years; 9th grade, 14 to 15 years old; 8th grade, 13 to 14; 7th grade, 12 to 13 years; and 6th grade, 11 to 12 years old.

The routine vaccination period ended in and before FY 2022 for the female individuals born in and before BFY 2006. However, those born in and after BFY 2007 are still targets for routine vaccination after FY 2022. Assuming that the vaccination coverages in FY 2022 for each BFY (Table 2 and Figure 1) are the same for each BFY after FY 2023, we estimated the cumulative vaccination coverage for BFYs 2007 to 2012 until FY 2028 (Table 3 and Figure 2).

**Table 3.** Estimated Routine HPV Vaccination With First-Dose Coverage Through Fiscal Year 2028 by Birth Fiscal Year

Grade^a^	Vaccination coverage, routine HPV vaccinations with a first dose, No. (%)																		
	Vaccination generation^b^						Vaccine-suspension generation^c^				Generation that received information individually^d^						Vaccine-resumed generation^e^		
	1994	1995	1996	1997	1998	1999	2000	2001	2002	2003	2004	2005	2006	2007	2008	2009	2010	2011	2012
11th	142 613 (23.65)	5855 (1.01)	NA	NA	NA	NA	NA	NA	NA	NA	NA	NA	NA	NA	NA	NA	NA	NA	NA
10th	178 852 (29.66)	362 112 (62.27)	39 224 (6.66)	5722 (0.98)	272 (0.05)	256 (0.04)	560 (0.10)	1366 (0.24)	2614 (0.47)	6774 (1.24)	49 015 (9.18)	104 516 (20.31)	85 413 (16.21)	85 350 (16.21)^f^	85 096 (16.21)^f^	83 513 (16.21)^f^	83 162 (16.21)^f^	82 046 (16.21)^f^	80 740 (16.21)^f^
9th	NA	64 387 (11.07)	351 268 (59.65)	52 608 (9.00)	3838 (0.66)	614 (0.11)	1112 (0.19)	534 (0.09)	800 (0.14)	1668 (0.31)	3944 (0.74)	14 364 (2.79)	36 634 (6.95)	52 802 (10.03)	52 645 (10.03)^f^	51 665 (10.03)^f^	51 448 (10.03)^f^	50 758 (10.03)^f^	49 950 (10.03)^f^
8th	NA	NA	70 362 (11.95)	345 946 (59.19)	71 102 (12.14)	13 198 (2.28)	1344 (0.23)	294 (0.05)	154 (0.03)	384 (0.07)	792 (0.15)	1838 (0.36)	6514 (1.24)	23 804 (4.52)	40 613 (7.74)	39 857 (7.74)	39 689 (7.74)^f^	39 157 (7.74)^f^	38 533 (7.74)^f^
7th	NA	NA	NA	60 149 (10.29)	380 434 (64.95)	374 526 (64.74)	70 888 (12.34)	1413 (0.25)	857 (0.15)	490 (0.09)	663 (0.12)	1334 (0.26)	3833 (0.73)	10 644 (2.02)	24 082 (4.59)	32 710 (6.35)	32 572 (6.35^f^	32 135 (6.35)^f^	31 623 (6.35)^f^
6th	NA	NA	NA	NA	4364 (0.75)	6836 (1.18)	6746 (1.17)	5010 (0.89)	236 (0.04)	178 (0.03)	96 (0.02)	134 (0.03)	402 (0.08)	908 (0.17)	3198 (0.61)	9438 (1.83)	14 494 (2.83)	14 300 (2.83)^f^	14 072 (2.83)^f^
Total	321 465 (53.31)	432 354 (74.36)	460 854 (78.25)	464 425 (79.47)	460 010 (78.53)	395 430 (68.36)	80 650 (14.04)	8617 (1.53)	4661 (0.84)	9494 (1.74)	54 510 (10.20)	122 186 (23.74)	132 796 (25.21)	173 508 (32.96)^f^	205 634 (39.18)^f^	217 183 (42.16)^f^	221 365 (43.16)^f^	218 396 (43.16)^f^	214 918 (43.16)^f^

Abbreviations: HPV, human papillomavirus; NA, not applicable.

^a^ Age ranges for grades are as follows: 11th grade, 16 to 17 years; 10th grade, 15 to 16 years; 9th grade, 14 to 15 years old; 8th grade, 13 to 14; 7th grade, 12 to 13 years; and 6th grade, 11 to 12 years old.

^b^ Birth fiscal yearly totals are as follows: 1994, 602 982 participants; 1995, 581 474 participants; 1996, 588 922 participants; 1997, 584 438 participants; 1998, 585 744 participants; and 1999, 578 486 participants. The total vaccination rate for this generation is 2 534 538 of 3 522 046 participants (71.96%; 95% CI, 71.92%-72.01%).

^c^ Birth fiscal yearly totals are as follows: 2000, 574 526 participants; 2001, 563 968 participants; 2002, 554 096 participants; and 2003, 544 934 participants. The total vaccination rate for this generation is 103 422 of 2 237 524 participants (4.62%; 95% CI, 4.59%-4.65%).

^d^ Birth fiscal yearly totals are as follows: 2004, 534 211 participants; 2005, 514 615 participants; 2006, 526 834 participants; 2007, 526 445 participants; 2008, 524 881 participants; and 2009, 515 115 participants. The total vaccination rate for this generation is 905 817 of 3 142 101 participants (28.83%; 95% CI, 28.78%-28.88%).

^e^ Birth fiscal yearly totals are as follows: 2010, 512 949 participants; 2011, 506 066 participants; and 2012, 498 009 participants. The total vaccination rate for this generation is 654 679 of 1 517 024 participants (43.16%; 95% CI, 43.08%-43.23%).

^f^ Denotes estimated values.

The estimated vaccination coverage to be achieved by FY 2028 was as follows: 32.96% (95% CI, 32.83%-33.10%) for BFY 2007, 39.18% (95% CI, 39.05%-39.31%) for BFY 2008, 42.16% (95% CI, 42.03%-42.30%) for BFY 2009, and 43.16% (95% CI, 43.02%-43.29%) for BFYs 2010 to 2012 (Figure 2). When assuming that the vaccination coverage in FY 2022 for each BFY was the same for each BFY from FY 2023 onward, the value plateaued at 43.16%.

The generation-specific vaccination coverage was 28.83% (95% CI, 28.78%-28.88%) for the generation that received information individually, which was 1.8 times higher than the vaccination coverage by FY 2022 of 16.16% (95% CI, 16.12%-16.20%), indicating a significant increase. However, this was less than one-third of the WHO target value of 90%. In addition, the vaccination coverage of the vaccine-resumed generation was 43.16% (95% CI, 43.08%-43.23%), which was significantly higher than that of the generation that received information individually but approximately one-half of the WHO target value.

Under the aforementioned assumption, we calculated the cumulative vaccination coverage for FY 2028 using increments of 1.2, 1.1, 0.9, and 0.8 multiples of the current vaccination coverage for each BFY (eFigure 2 in the Supplement). This analysis revealed the degree to which HPV vaccination coverage would reach a steady state under the current trends for Japanese vaccination coverage. Depending on the multiplier, a small range in future vaccination coverage rates was estimated, but none was in line with the 90% WHO goal and was even lower than what was achieved for the vaccination generation.

## Discussion

In this cross-sectional study, we calculated the BFY-specific vaccination coverage for each FY, the cumulative first-dose coverage for each BFY, and the generation-specific vaccination coverage in Japan, where HPV vaccination policies have changed greatly. The results showed that HPV vaccination coverage among Japanese women remained low and that even the cumulative first-dose coverage of each BFY, which was to be achieved by FY 2028, would not reach the WHO target value.

The multiple media reports of potentially serious, diverse symptoms after HPV vaccination and the suspension of proactive governmental recommendations for the HPV vaccine in June 2013 had serious and long-lasting negative outcomes for HPV vaccine acceptance among target female individuals and their parents and/or guardians. Consequently, the HPV vaccine continues to have a strong negative image in Japan.^44,45^

In Japan, the incidence of cervical cancer is rapidly increasing among young female individuals, and its prevention using adequate HPV vaccination is crucial.^46^ We proposed various vaccination coverage dissemination strategies to restore HPV vaccination coverage as soon as possible.^47,48^ However, they were never incorporated into the governmental policy.

In contrast, the high coverage for early childhood vaccination in Japan is notable. According to a 2017 to 2018 survey, coverages were generally acceptable, at 95.9% for the *Haemophilus influenzae* type b vaccine at age 3 months, 95.5% for the first dose of the pediatric pneumococcal vaccine at age 3 months, and 85.7% for the first dose of the quadruple vaccine (diphtheria, pertussis, tetanus, and typhoid) at age 3 months.^49^ In the context of the COVID-19 vaccine, the reported vaccination coverage of at least 1 dose among 12- to 19-year-olds was 66.9%, which is surprisingly similar to the 70% HPV coverage of the HPV vaccination generation.^50^

The good vaccination coverage of other childhood vaccines and COVID-19 vaccines during the COVID-19 pandemic in Japan supports the fact that vaccination coverage of HPV vaccines is unique. However, some countries that experienced a nationwide decline in vaccination coverage due to negative information about the HPV vaccine have subsequently recovered their vaccination coverage. In Ireland, where HPV vaccination coverage also declined by approximately 30% due to media reports of potentially serious, diverse symptoms after HPV vaccination and antivaccine group campaigns, 35 different organizations worked together to achieve approximately 10% improvement in the following year.^51^ Accordingly, MHLW should recognize the HPV vaccination coverage situation as a crisis and implement a campaign similar to that in Ireland. Furthermore, a nationwide HPV vaccination database must be established, and more rigorous efforts must be taken to overcome HPV vaccine hesitancy among the public for both male and female individuals. We also suggest strengthening cervical cancer screening recommendations, extending subsidized vaccinations to teenage boys, and introducing HPV testing based on the timely establishment of local government systems.

In 2015, the WHO criticized the Japanese government’s policy of suspension of the proactive recommendation of HPV vaccines, stating that “policy decisions based on weak evidence, leading to lack of use of safe and effective vaccines, can result in real harm.”^12^ Notably, the current status of vaccination coverage has not been adequately restored, and this is already negatively impacting women in Japan, resulting in greatly increased HPV-caused precancerous cervical lesions.^34^ The warning by the WHO has truly become a reality in Japan, and the increase in cervical cancer cases in the future is of great concern.

### Strengths and Limitations

The strength of this study is that it demonstrated Japan’s HPV vaccination coverage using the latest national data, enabling us to analyze vaccine uptake trends and estimate a serious shortcoming in coverage recovery as far forward as FY 2028. This study also has some limitations. First, this study does not include more recent vaccination coverage for FY 2023. Second, individuals who paid out-of-pocket for vaccination were not included in this study. Third, this study dealt with vaccination coverage across Japan, and regional differences were not analyzed. Furthermore, the type of vaccine and HPV status at the time of initiation of vaccination are unknown.

## Conclusions

HPV vaccination coverage, even after the resumption of MHLW’s proactive recommendations, has recovered only minimally in Japan. For the first time, we have ascertained the cumulative HPV vaccination coverage with the first dose by BFY through FY 2022, enabling us to plan appropriately for the future of cervical cancer controls. The vaccination coverage to be achieved by FY 2028 was estimated to be below the WHO targets. Stronger cervical cancer control measures are required, particularly for the vaccine-resumed generation, which will plateau at approximately one-half the WHO target values.

## Notes

Supplement. **eTable 1.** Number of routine human papillomavirus (HPV) vaccinations with the first dose by age at vaccination, as disclosed by the MHLW **eTable 2.** Correspondence chart between age at vaccination, school grade, and BFY (eg, FY 2022) **eFigure 1.** Correspondence chart between calendar year and fiscal year **eFigure 2.** Fluctuation in the cumulative vaccination coverages until the end of BFY 2028 resulting from the changes in the vaccination coverages from BFY 2023 onward

Supplement 2.
Data Sharing Statement
