# Supplementary material for: Human Papillomavirus Vaccination by Birth Fiscal Year in Japan
Source: JAMA Netw Open. 2024 Jul 16;7(7):e2422513. doi: 10.1001/jamanetworkopen.2024.22513 (PMC11252895; doi:10.1001/jamanetworkopen.2024.22513)
Supplement: Supplement. — eTable 1. Number of routine human papillomavirus (HPV) vaccinations with the first dose by age at vaccination, as disclosed by the MHLW eTable 2. Correspondence chart between age at vaccination, school grade, and BFY (eg, FY 2022) eFigure 1. Correspondence chart between calendar year and fiscal year eFigure 2. Fluctuation in the cumulative vaccination coverages until the end of BFY 2028 resulting from the changes in the vaccination coverages from BFY 2023 onward [file jamanetwopen-e2422513-s001.pdf]

Supplemental Online Content

Yagi A, Ueda Y, Oka E, Nakagawa S, Kimura T. Human papillomavirus vaccination by birth fiscal year in Japan. *JAMA Netw Open*. 2024;7(7):e2422513. doi:10.1001/jamanetworkopen.2024.22513

eTable 1. Number of routine human papillomavirus (HPV) vaccinations with the first dose by age, as disclosed by the MHLW

eTable 2. Correspondence chart between age at vaccination, school grade, and BFY (e.g., FY 2022)

eFigure 1. Correspondence chart between calendar year and fiscal year

eFigure 2. Fluctuation in the cumulative vaccination coverages until the end of BFY 2028 resulting from the changes in the vaccination coverages from BFY 2023 onward

This supplemental material has been provided by the authors to give readers additional information about their work.

© 2024 Yagi A et al. *JAMA Network Open*.

eTable 1. Number of routine human papillomavirus (HPV) vaccinations with the first dose by age, as disclosed by the MHLW

Age at vaccination	Vaccinations per fiscal year (FY)												
	2010	2011	2012	2013	2014	2015	2016	2017	2018	2019	2020	2021	2022
17 y/o	—	71,307	2,928	—	—	—	—	—	—	—	—	—	—
16 y/o	178,852	252,362	22,539	2,861	136	128	280	683	1,307	3,387	29,436	45,150	36,939
15 y/o	64,387	356,690	45,916	4,780	443	684	547	1,083	2,141	5,359	26,761	77,683	72,439
14 y/o	70,362	348,607	61,855	8,518	979	703	344	592	1,230	2,891	10,439	30,219	44,577
13 y/o	60,149	363,190	222,814	50,552	1,324	618	338	557	1,090	2,869	8,579	23,943	38,711
12 y/o	4,364	193,635	190,636	29,440	879	475	277	365	841	2,337	6,921	16,760	28,072
11 y/o	—	3,418	3,373	2,505	118	89	48	67	201	454	1,599	4,719	5,705

y/o: years old

© 2024 Yagi A et al. *JAMA Network Open*.

eTable 2. Correspondence chart between age at vaccination, school grade, and BFY (e.g., FY 2022)

Age at Vaccination	School Grade	BFY
11 y/o	6th	2010
12 y/o	6th, 7th	2010, 2009
13 y/o	7th, 8th	2009, 2008
14 y/o	8th, 9th	2008, 2007
15 y/o	9th, 10th	2007, 2006
16 y/o	10th	2006

y/o: years old

© 2024 Yagi A et al. *JAMA Network Open*.

Supplementary Figure 1. Correspondence chart between calendar year and fiscal year

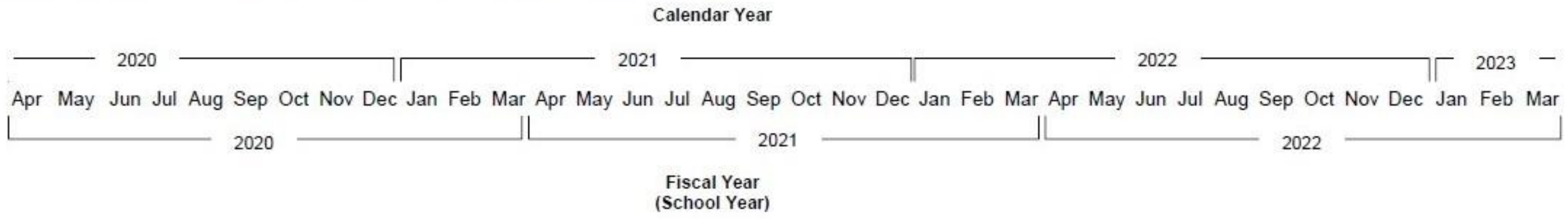

© 2024 Yagi A et al. *JAMA Network Open*.

Supplementary Figure 2. Fluctuation in the cumulative vaccination coverages until the end of BFY 2028 resulting from the changes in the vaccination coverages from BFY 2023 onwards

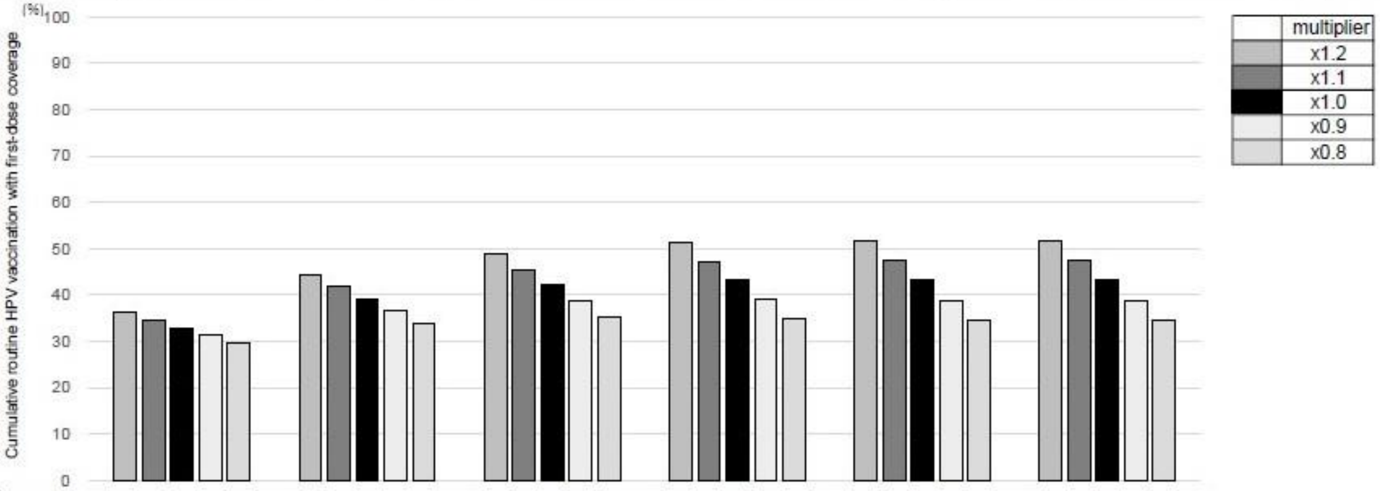

BFY	2007	2008	2009	2010	2011	2012
x1.2	36.19	44.43	48.96	51.23	51.80	51.80
x1.1	34.57	41.8	45.55	47.19	47.47	47.47
x1.0	32.95	39.18	42.16	43.16	43.16	43.16
x0.9	31.33	36.56	38.77	39.14	38.86	38.86
x0.8	29.71	33.93	35.36	35.09	34.52	34.52

We calculated the cumulative vaccination coverage until FY 2028 using increments of 1.2, 1.1, 0.9, and 0.8 multiples of the current vaccination coverage for each BFY.

© 2024 Yagi A et al. *JAMA Network Open*.
