# Supplementary material for: Human Papillomavirus Vaccination by Birth Fiscal Year in Japan
Source: JAMA Netw Open. 2024 Jul 16;7(7):e2422513. doi: 10.1001/jamanetworkopen.2024.22513 (PMC11252895; doi:10.1001/jamanetworkopen.2024.22513)
Supplement: Supplement 2. — Data Sharing Statement [file jamanetwopen-e2422513-s002.pdf]

Data Sharing Statement

Yagi. Human Papillomavirus Vaccination by Birth Fiscal Year in Japan. *JAMA Netw Open*. Published July 16, 2024. doi:10.1001/jamanetworkopen.2024.22513

Data
Data available: Yes
Data types: Data dictionary
How to access data: https://www.mhlw.go.jp/file/05-Shingikai-10601000-Daijinkanboukouseikagakuka-Kouseikagakuka/0000147016.pdf, https://www.e-stat.go.jp/, https://www.stat.go.jp/data/kokusei/2020/index.html
When available: beginning date: 07-01-2024

Supporting Documents
Document types: None

Additional Information
Who can access the data: anyone requesting the data
Types of analyses: any purpose
Mechanisms of data availability: without investigator support
Any additional restrictions: NA
